# Two New Steroidal Saponins with Potential Anti-Inflammatory Effects from the Aerial Parts of *Gnetum formosum* Markgr.

**DOI:** 10.3390/plants13152100

**Published:** 2024-07-29

**Authors:** Ngo Van Hieu, Le Ba Vinh, Nguyen Viet Phong, Pham Van Cong, Nguyen Tien Dat, Nguyen Van Dan, Ngo Viet Duc, Hoang Minh Tao, Le Thi Tam, Le Tuan Anh, Nguyen Cao Cuong, Bui Huu Tai, Seo Young Yang, Hoang Le Tuan Anh

**Affiliations:** 1 Center for High Technology Research and Development, Vietnam Academy of Science and Technology (VAST), Hanoi 10072, Vietnam; ngohieu03041997@gmail.com (N.V.H.); phamcong1990@gmail.com (P.V.C.); ngtiend@gmail.com (N.T.D.); nvdan100@gmail.com (N.V.D.); ngovietduc.cretech@gmail.com (N.V.D.); htm1205@gmail.com (H.M.T.); lamtamhoaduoc@gmail.com (L.T.T.); 2 Graduate University of Science and Technology, Vietnam Academy of Science and Technology (VAST), Hanoi 10072, Vietnam; 3 Institute of Marine Biochemistry, Vietnam Academy of Science and Technology (VAST), Hanoi 10072, Vietnam; vinhrooney@gmail.com (L.B.V.); ngvietphong@gmail.com (N.V.P.); bhtaiich@gmail.com (B.H.T.); 4 Department of Biology Education, Teachers College and Institute for Phylogenomics and Evolution, Kyungpook National University, Daegu 41566, Republic of Korea; 5 Vietnam National Museum of Nature, Vietnam Academy of Science and Technology (VAST), Hanoi 10072, Vietnam; tasa207@gmail.com; 6 Faculty of Medicine and Pharmacy, Yersin University, Da Lat 66100, Vietnam; nguyencaocuong2712@gmail.com

**Keywords:** *Gnetum formosum* Markgr., steroidal saponin, gnetumoside A, gnetumoside B, anti-inflammatory

## Abstract

*Gnetum formosum* Markgr., a member of the Gnetaceae family, is distributed in Vietnam. This plant remains a botanical enigma with an unexplored diversity of chemical constituents and pharmacological effects. In this study, two new steroidal saponins, namely gnetumosides A (**1**) and B (**2**), were isolated from the aerial parts of *G. formosum*. Their chemical structures were elucidated using spectroscopic techniques, including high-resolution electrospray ionization mass spectrometry (HR-ESI-MS) and NMR, along with chemical hydrolysis and comparison with the reported literature. The potential anti-inflammatory effects of the isolated compounds were evaluated by measuring lipopolysaccharide-stimulated nitric oxide (NO) production in murine macrophage cells. Notably, compound **1** exhibited the most potent inhibitory activity (IC_50_ = 14.10 ± 0.75 µM), comparable to dexamethasone. Additionally, the mechanisms underlying the observed anti-inflammatory effects were investigated through molecular docking and molecular dynamics simulations on inducible nitric oxide synthase (iNOS) and cyclooxygenase-2 (COX-2) proteins. This study is the first to investigate the chemical constituents and pharmacological effects of *G. formosum*.

## 1. Introduction

Inflammation, characterized by a complex interplay of molecular pathways, is a vital biological response to various stimuli, such as injury or infection [1]. In pathological conditions, such as inflammation, nitric oxide is produced with the participation of the inducible form of NO synthase (iNOS). The production of large amounts of nitric oxide (NO) can lead to the excessive activation of cyclooxygenase (COX), resulting in the formation of large amounts of pro-inflammatory prostaglandins and reactive oxygen species [2]. The intricate roles of these mediators in modulating inflammatory responses have garnered significant scientific interest, driving research to understand their mechanisms and potential therapeutic implications. The murine macrophage RAW264.7 cell line stands as a valuable model system for investigating inflammation owing to its robust responsiveness to inflammatory stimuli, offering insights into the underlying cellular and molecular mechanisms [3]. Natural products derived from diverse sources, such as botanicals, marine life, and microbes, offer promising solutions for treating inflammation due to their safety, affordability, and efficacy [4,5]. These compounds exhibit a wide range of pharmacological activities, including anti-inflammatory properties, making them valuable for developing novel therapeutics [6,7]. Their long history of use in traditional medicine systems without significant adverse effects underscores their safety profile [8]. Additionally, their cost-effectiveness compared to synthetic drugs enables broader access to treatment, especially in resource-limited settings [9]. Moreover, natural products have demonstrated remarkable efficacy in both preclinical and clinical studies, targeting multiple points in the inflammatory pathway [10,11].

The genus *Gnetum* (Gnetaceae) comprises approximately 40 species. They are typically found in the tropical and subtropical regions of Asia, southwestern Africa, and South America. Many *Gnetum* species are edible, with seeds often roasted and foliage used as a leaf vegetable. The family Gnetaceae is renowned for being a rich source of plant-derived stilbenoids and saponins [12]. *Gnetum formosum*, a member of the Gnetaceae family, remains a botanical enigma with an unexplored diversity of chemical constituents and pharmacological effects. Moreover, the roots and stems of this plant have been employed in Vietnamese traditional medicine to alleviate pain, address postpartum conditions, and counteract the effects of toxins, including snakebites [13].

Molecular docking simulation and dynamics play a crucial role in drug development by enabling researchers to predict drug candidates’ binding affinity and stability with their target proteins [14]. Compared to traditional experimental methods, these techniques are fast, efficient, and cost-effective. They facilitate the high-throughput screening of large compound libraries, significantly accelerating drug discovery. Additionally, they offer detailed insights into molecular interactions, aiding in the rational design of more effective and selective drugs [15].

**Figure 1:**
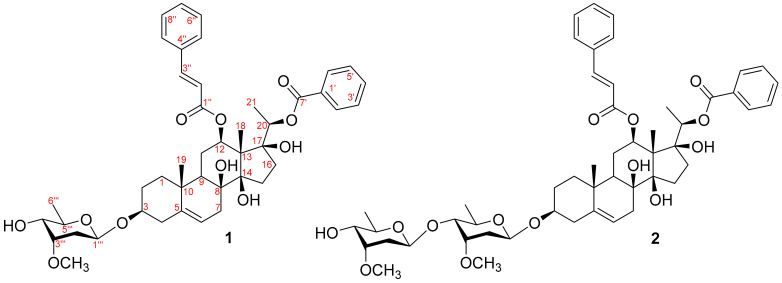
Structures of the steroid glycosides isolated (**1** and **2**) from *G. formosum*.

As part of our ongoing research on Vietnamese medicinal plants with anti-inflammatory properties, this study investigates the potential anti-inflammatory activities of secondary metabolites from the aerial parts of *G. formosum* [16]. This study represents the first report on chemical constituents isolated from the aerial parts of *G. formosum*, identifying their potential anti-inflammatory activities. Two new compounds (**1** and **2**) were isolated from *G. formosum* (Figure 1). Their structures were determined using spectroscopic analysis, including high-resolution electrospray ionization mass spectrometry (HR-ESI-MS) and NMR, as well as comparison with known data. The potential anti-inflammatory activities of the isolated compounds were evaluated by measuring lipopolysaccharide (LPS)-stimulated NO production in murine macrophage cells. Notably, compound **1** exhibited the most potent inhibitory activity (IC_50_ = 14.5 µM). Additionally, the mechanisms underlying the resulting anti-inflammatory effects were explored through molecular docking and molecular dynamics (MD) studies on iNOS and COX-2 proteins.

## 2. Results and Discussion

**Figure 2:**
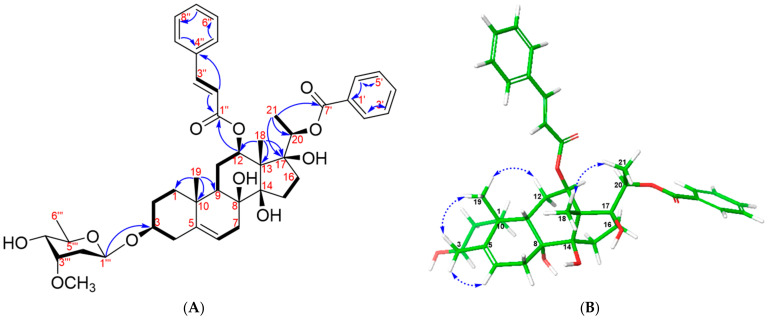
(**A**) Selected HMBC and ^1^H-^1^H COSY cross-peaks and (**B**) key NOESY correlations of the aglycon of compound **1**.

Compound **1** was obtained as a white amorphous powder with ${\left[\alpha \right]}_{D}^{20}$ + 36.77 (*c* 0.05, MeOH) (Figures S1–S7). Its molecular formula C_44_H_56_O_11_ was identified using HR-ESI-MS, where a protonated adduct exhibited a molecular ion peak at *m*/*z* 761.3909, consistent with [M + H]^+^ (calcd. 761.3895 for C_44_H_57_O_11_^+^; error: 0.0014). The ^1^H-NMR and HSQC spectra of compound **1** (600 MHz, MeOD-*d*_4_) showed signals typical of a benzoyl group [*δ*_H_ 7.96 (dd, *J* = 1.2, 8.4 Hz, H-2′/H-6′), 7.35 (overlapped, H-3′/H-5′), and 7.55 (1H, m, H-4′)] and a cinnamoyl group [*δ*_H_ 6.07 (1H, d, *J* = 16.2 Hz, H-2″), 7.35 (1H, d, *J* = 16.2 Hz, H-3″), 7.24 (1H, t, *J* = 1.2, 8.4 Hz, H-5″/H-9″), 7.34 (1H, overlapped, H-6″/H-8″), and 7.38 (1H, m, H-7″)] [17]. Additionally, the ^1^H-NMR spectrum of compound **1** displayed the presence of a methoxy group [*δ*_H_ 3.43 (3H, s, -OCH_3_)], an olefinic proton [*δ*_H_ 5.35 (1H, m, H-6)], and an anomeric proton [*δ*_H_ 4.85 (1H, m)] (Table 1). Furthermore, the ^1^H NMR data of compound **1** exhibited the signals of three tertiary methyl groups at *δ*_H_ 1.64 (3H, s, H-18), 1.12 (3H, s, H-19), and 1.33 (3H, d, *J* = 6.0 Hz, H-21), correlating with their respective carbon signals in the HSQC spectrum at *δ*_C_ 11.3, 18.5, and 15.2. The ^13^C-NMR, HSQC, and HMBC spectra of compound **1** confirmed the presence of forty-three carbon peaks, including twenty-one carbon signals for the steroid aglycon, one benzoyl group, one cinnamoyl group, and six carbons for the sugar moiety. The chemical shifts of the aglycon of compound **1** were similar to those of stemucronatoside A [17]. The absolute configuration of the sugar moiety was identified as *β*-D-cymaropyranosyl through acid hydrolysis and further confirmed by the optical rotation value [18,19,20]. The HMBC cross-peak of anomeric H-1_Cym_ (*δ*_H_ 4.85) with C-3 (*δ*_C_ 79.3) was clearly observed (Figure 2A). Moreover, the strong HMBC signals of H-12 (*δ*_H_ 4.86) with C-1″ (*δ*_C_ 168.1) indicated the linkage of the cinnamoyl group to the C-12 position [21]. Similarly, the HMBC cross-peaks of H-20 (*δ*_H_ 4.81) with C-7′ (*δ*_C_ 167.0) were unambiguously identified, confirming the attachment of the benzoyl group to the C-20 position [21]. The relative configuration of compound **1** was determined through NOESY correlations analysis, demonstrating similarity with that of stemucronatoside A [17] (Figure 2B). Thus, compound **1** was identified as 12-*β*-*O*-cinnamoyl-20-*O*-benzoylsarcostin-3-*O*-*β*-d-cymaropyranoside and was named gnetumoside A.

Compound **2** was obtained as a white amorphous powder with ${\left[\alpha \right]}_{D}^{20}$ + 29.45 (*c* 0.06, MeOH) (Figures S8–S14). The HR-ESI-MS data of compound **2** revealed a protonated molecular ion peak [M + H]^+^ at *m/z* 905.4694, corresponding to a molecular formula of C_51_H_68_O_14_ (calcd. for C_51_H_69_O_14_^+^, 905.4682, error: 0.0012). The NMR data of the aglycon of compound **2** resembled those of compound **1**, suggesting a structural similarity of a steroid glycoside between the two compounds (Table 1). In addition, the ^1^H-NMR spectrum of compound **2** showed signals typical of two anomeric protons at *δ*_H_ 4.89 (1H, m, H-1_CymI_) and 4.80 (1H, dd, *J* = 1.8, 9.6 Hz, H-1_CymII_). These resonances had HSQC correlations with the corresponding anomeric carbons at *δ*_C_ 97.2 (C-1_CymI_) and 101.2 (C-1_CymII_), indicating the presence of two sugar moieties in the chemical structure of compound **2**. The configuration of *β*-d-cymaropyranosyl moieties was confirmed through acid hydrolysis and the optical rotation value. Moreover, HMBC correlations were observed from *δ*_H_ 4.80 (1H, dd, *J* = 1.8, 9.6 Hz, H-1_CymII_) to C-4_CymI_ and from *δ*_H_ 4.89 (1H, m, H-1_CymI_) to C-3, indicating the connection of the disaccharides to C-3 of the aglycone. Thus, compound **2** was identified as 12-*β*-*O*-cinnamoyl-20-*O*-benzoylsarcostin-3-*O*-*β*-d-cymaropyranosyl-(1→4)-*β*-d-cymaropyranoside and was named gnetumoside B.

NO, a widely present natural compound, is known to play roles in both normal bodily functions and disease processes. Notably, excessive NO production has been linked to various medical conditions, including diabetes, circulatory shock, atherosclerosis, cancer, and chronic inflammatory diseases [22]. Therefore, controlling NO secretion is a crucial pharmacological approach in drug research. To assess the safety profile of the compounds, the MTT assay was initially conducted to determine non-toxic concentrations [6]. As a result, none of the compounds showed toxicity even at a concentration of 100 µg/mL. Subsequently, the anti-inflammatory properties of compounds **1** and **2** were examined by evaluating their inhibitory effects on NO production. Compound **1** exhibited the highest inhibitory activity, with an IC_50_ value of 14.10 ± 0.75 μM, similar to that of the positive control, dexamethasone (IC_50_ = 13.35 ± 1.52 μM). On the other hand, compound **2** exhibited moderate inhibition of NO production, with an IC_50_ value of 27.88 ± 0.86 μM. The results are presented as the mean ± standard deviation of triplicate individual experiments (*n* = 3). Based on these findings, compound **1** was selected for further investigation into its underlying mechanisms in inflammation.

**Figure 3:**
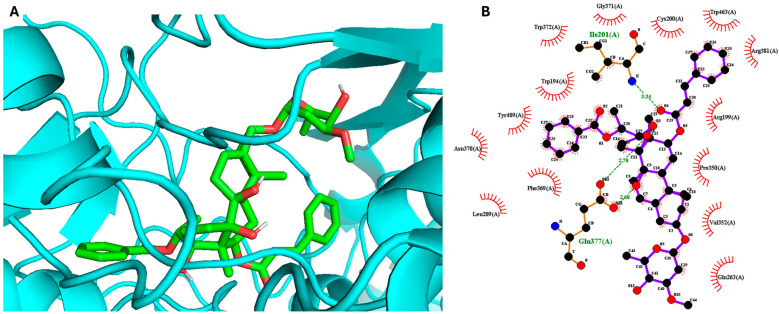
Molecular docking analysis of compound **1** for iNOS inhibition. (**A**) Three-dimensional docking poses and (**B**) two-dimensional interaction diagrams showing the interaction of compound **1** with the binding site of iNOS.

**Figure 4:**
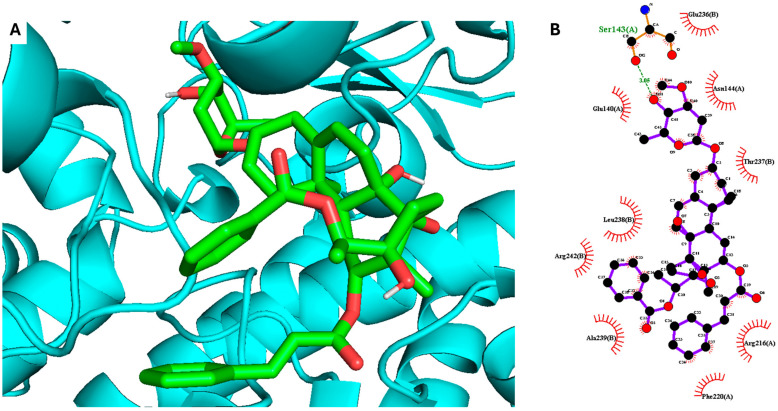
Molecular docking analysis of compound **1** for COX-2 inhibition. (**A**) Three-dimensional docking poses and (**B**) two-dimensional interaction diagrams showing the interaction of compound **1** with the binding site of COX-2.

The most active compound **1**, gnetumoside A, was docked into the active sites of the iNOS and COX-2 proteins, aiming to uncover the binding sites, crucial interactions, and molecular mechanisms underlying its anti-inflammatory action [23]. The crystalline structures of iNOS (4NOS) and COX-2 (5KIR) were obtained from the Protein Data Bank. The results indicated that compound **1** can bind to the active sites of both iNOS and COX-2, with docking scores of −11.5 and −8.7 kcal/mol, respectively. This underscores its potential anti-inflammatory effect. The ester group of compound **1** established hydrogen bond interactions with GLU377 (2.68, 2.78) and ILE301 (3.34) in the active site of the iNOS protein (Figure 3). Meanwhile, SER143 (3.05) was found to play a crucial role in inducing and stabilizing the active conformation of the COX-2 protein (Figure 4).

**Figure 5:**
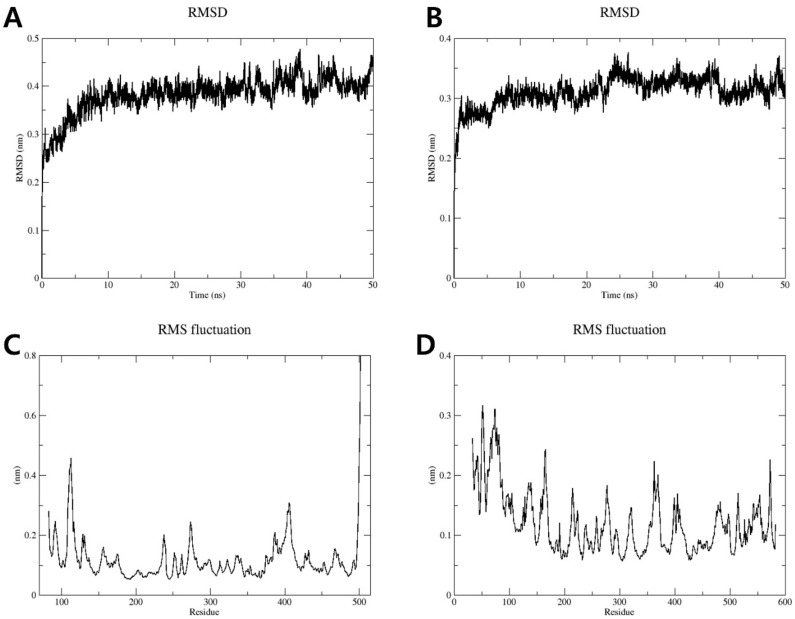
Molecular dynamics analyses of the interactions between compound **1** and the proteins iNOS and COX-2. RMSD of the protein backbones in the complexes formed by compound **1** with (**A**) iNOS and (**B**) COX-2. RMSF of the amino acid residues in the complexes formed by compound **1** with (**C**) iNOS and (**D**) COX-2.

To further examine the conformational stability of and variations in the complexes formed by compound **1** with iNOS and COX-2 proteins, MD simulations were performed using GROMACS 2022.1 for a 50 ns trajectory. As shown in Figure 5A,B, the root mean square deviation (RMSD) plots for the complexes formed by compound **1** with the iNOS and COX-2 proteins illustrate the structural conformation changes over the 50 ns simulation period. The RMSD values rose to approximately 0.4 nm and 0.3 nm for the iNOS–compound **1** and COX-2–compound **1** complexes, respectively, indicating initial conformational adjustments before stabilizing. This suggests that the target proteins reach a stable conformation after binding compound **1**, demonstrating that compound **1** does not cause significant structural destabilization.

The root mean square fluctuation (RMSF) plots (Figure 5C,D) indicate the flexibility of specific residues within the iNOS and COX-2 proteins. Peaks up to 0.6–0.8 nm in the iNOS–compound **1** complex and up to 0.3 nm in the COX-2–compound **1** complex indicate regions of higher flexibility, which are often associated with loop regions or terminal residues. In contrast, residues within the binding sites of compound **1** with iNOS and COX-2 exhibit RMSF values below 0.2 nm, indicating the stability of these regions.

**Figure 6:**
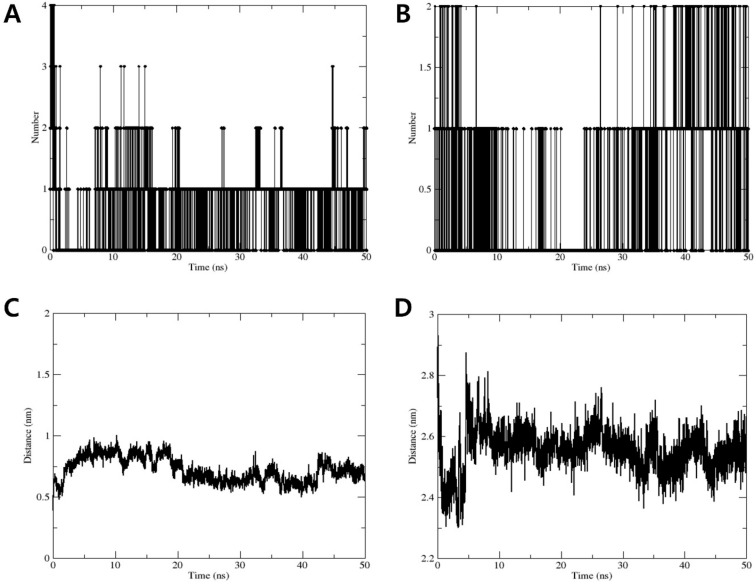
Number of hydrogen bonds in the complex formed by compound **1** with (**A**) iNOS and (**B**) COX-2. The average distances of compound **1** from (**C**) iNOS and (**D**) COX-2.

Additionally, the number of hydrogen bonds between compound **1** and the iNOS and COX-2 proteins was tracked over the MD simulation period. The results (Figure 6A,B) indicated that while the number of hydrogen bonds fluctuates, it remains consistent overall, suggesting stable interactions between compound **1** and the target proteins. Distance–time plots (Figure 6C,D) track the distance between specific atoms or functional groups of compound **1** and key residues of iNOS and COX-2. The relatively stable distance values (0.5–0.9 nm for the iNOS–compound **1** complex and 2.3–2.7 nm for the COX-2–compound **1** complex) suggest persistent interactions, such as hydrogen bonds, hydrophobic interactions, or ionic interactions. Occasional fluctuations may indicate minor conformational adjustments or transient interactions but do not significantly affect the overall binding stability.

**Figure 7:**
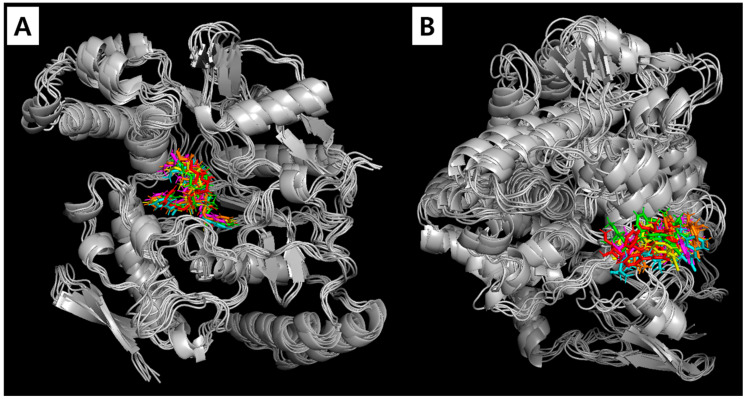
Superposition of compound **1** in the active sites of iNOS (**A**) and COX-2 (**B**).

Figure 7 illustrates the superposition of compound **1** in the active sites of iNOS and COX-2 during the MD simulations. No significant changes were observed throughout the 50 ns MD trajectory, indicating the stability of both the iNOS–compound **1** and COX-2–compound **1** complexes. Additionally, the MD results, when compared with the enzyme kinetics and molecular docking outcomes, demonstrated a consistent inhibition mode and consistent binding interactions across all experiments. These findings underscore the reliability and validity of the MD simulation approach employed in this study.

## 3. Materials and Methods

### 3.1. General Experimental Procedures

Optical rotations, one-dimensional (1D) and two-dimensional (2D) NMR, and HR-ESI-MS were recorded using the instruments P-2000 (JASCO, Tokyo, Japan), AVANCE NEO 600 FT-NMR (Bruker, Billerica, MA, USA), and 6530 Accurate-Mass spectrometer (Agilent Technologies, Santa Clara, CA, USA), respectively. Silica gel 60 (230–400 mesh; Merck, Germany), Sephadex LH-20 (GE Healthcare Bio-Science AB, Uppsala, Sweden), and Diaion HP-20 (Supelco, Bellefonte, PA, USA) resins were used for open column chromatography (CC). Additionally, planar chromatography (TLC) was conducted using pre-coated silica gel 60 F254 and RP-18 F254S plates (0.25 mm, Merck, Darmstadt, Germany). Spots were visualized under UV light at 254 nm and 365 nm wavelengths, as well as with 10% H2SO4, followed by heating for 3–5 min. All chemical substances were sourced from Sigma-Aldrich (St. Louis, MO, USA).

### 3.2. Botanical Specimens

The aerial parts of *G. formosum* Markgr. were collected from Quang Tri, Vietnam, in October 2022 and taxonomically identified by Dr. Le Tuan Anh, one of the authors. The specimen (GF2022) was deposited at the Center for High Technology Research and Development, Vietnam Academy of Science and Technology, Vietnam.

### 3.3. Extraction and Isolation

The dried upper parts of *G. formosum* (4.0 kg) were cut into 1 cm pieces and soaked in 99.5% MeOH (10 L × 3 times) at room temperature. The resulting methanol solution was evaporated using a rotary evaporator to obtain a MeOH extract residue (300 g). This residue was then dissolved in water and sequentially partitioned with *n*-hexane (H), dichloromethane (CH_2_Cl_2_), and ethyl acetate (EtOAc) to obtain *n*-hexane (100 g), CH_2_Cl_2_ (30 g), EtOAc (90 g), and aqueous (W) extracts, respectively. Based on TLC analysis, the *n*-hexane and CH2Cl2 fractions were found to contain significant amounts of fatty acids, while the water layer was rich in sugars. Consequently, EtOAc was chosen for further purification.

Next, the ethyl acetate fraction (90 g) was subjected to silica gel CC using a solvent mixture of CH_2_Cl_2_-MeOH-H_2_O (45:10:1, *v*/*v*/*v*) to obtain nine subfractions (E1 to E9). Subfraction E2 underwent additional fractionation using reversed-phase YMC RP-C18 silica gel, employing a gradient of acetone and water (1:3 to 3:1, *v*/*v*), yielding three subfractions (E2A to E2C). Based on TLC guidance, subfraction E2B (3.0 g) was purified using silica gel CC with a CH_2_Cl_2_-MeOH solvent system (200:1, *v*/*v*), followed by further purification on Sephadex LH-20 CC with MeOH-H_2_O (5:1, *v*/*v*), resulting in the isolation of compounds **1** (4.5 mg) and **2** (5.9 mg).

Gnetumoside A (**1**). White amorphous powder; ${\left[\alpha \right]}_{D}^{20}$ + 36.77 (*c* 0.05, MeOH); ^1^H (600 MHz in MeOD-*d_4_*) and ^13^C-NMR (150 MHz in MeOD-*d_4_*): see Table 1; HR-ESI-MS: *m/z* 761.3909 [M + H]^+^ (calcd. for C_44_H_57_O_11_^+^, 761.3895).

Gnetumoside B (**2**). White amorphous powder; ${\left[\alpha \right]}_{D}^{20}$ + 29.45 (*c* 0.06, MeOH), ^1^H (600 MHz in MeOD-*d_4_*) and ^13^C-NMR (150 MHz in MeOD-*d_4_*): see Table 1; HR-ESI-MS: *m/z* 905.4694 [M + H]^+^ (calcd. for C_51_H_69_O_14_^+^, 905.4682).

### 3.4. Acidic Decomposition and Analysis of Sugar Moieties’ Absolute Configuration

Compounds **1** and **2** (each 2 mg) were individually hydrolyzed by heating at 55 °C overnight in a mixture of 15% hydrochloric acid/EtOH (1:1, *v*/*v*) [4]. The sample was then partitioned with CH_2_Cl_2_ and water. The optical rotation of the monosaccharide was determined and compared with the corresponding reference. d-Cymarose was identified based on the optical rotation value, TLC analysis, and comparison with authentic sugar [R_f_ 0.51 in CHCl_3_–CH_3_OH (9:1), R_f_ 0.58 in CH_2_Cl_2_–C_2_H_5_OH (9:1), and R_f_ 0.41 in petroleum ether-acetone (3:2); ${\left[\alpha \right]}_{D}^{20}$ + 50.0 (*c* 0.4, H_2_O)] [18,19,20].

### 3.5. MTT and Measurement of NO Production

To assess cytotoxicity and cell viability, the MTT assay was performed on NO and RAW264.7 cells. Briefly, cells were seeded in 96-well plates at a density of 5000 cells per well and allowed to adhere overnight. After treatment with varying concentrations of the test substances, cells were incubated with MTT solution (0.5 mg/mL) for 4 h at 37 °C. The formazan crystals formed were solubilized using dimethyl sulfoxide (DMSO), and absorbance was measured at 540 nm using a microplate reader. Cell viability was calculated relative to untreated controls, and each experiment was performed in triplicate. Briefly, various concentrations (0.8, 4, 20, and 100 µg/mL) were tested with the cells for 4 h [3]. NO inhibitory activity was evaluated based on the Griess reaction, as described previously [6,24]. Absorbance levels were measured using a microplate reader at a wavelength of 540 nm. Dexamethasone was used as a positive control.

### 3.6. Molecular Docking Simulation

Molecular docking simulations were performed using AutoDock Vina 1.1.2, following the reported procedure [23]. The three-dimensional (3D) crystal structures of iNOS (PDB ID: 4NOS) [25] and COX-2 (PDB ID: 5KIR) [26] were obtained from the Protein Data Bank. The 3D structure of the active compound was generated and energy-minimized using Spartan’24 (Wavefunction, Irvine, CA, USA). The docking results were analyzed using PyMOL (Version 2.5.4, Schrodinger, LLC for education purposes). A 2D docking image was created using LigPlot+ v.2.2.8 [27].

### 3.7. Molecular Dynamics Simulation

MD simulations were performed using GROMACS 2022.1, following the reported procedure [28,29]. The CHARMM-GUI web platform and CHARMM36 force field were used for system setup [30]. A TIP3P explicit solvation model with periodic boundary conditions in a box was applied. Na^+^ and/or Cl^−^ ions were added to neutralize the system. Energy minimization was performed using a maximum force threshold of 10 kJ/mol. NVT equilibration at 300 K and NPT equilibration at 1.01325 bar were performed for a duration of 50 ns. The simulation data were analyzed using PyMOL 2.5.4 and Grace software version 5.0 (https://plasma-gate.weizmann.ac.il/Grace/).

## 4. Conclusions

Natural products derived from medicinal herbs are invaluable in the research and development of innovative medications, including those with anti-inflammatory, anti-allergic, antioxidant, antibacterial, and anticancer properties [31]. Notably, 60% of the newly approved small molecular medications in the past 30 years were derived from or linked to natural products [32]. Various approaches to evaluating the pharmacological effects of natural compounds have contributed substantially to discoveries in drug development [33]. Phytochemical analyses of *G. formosum* aerial parts led to the identification of two new compounds, gnetumosides A (**1**) and B (**2**). High-resolution mass spectrometry and 1D and 2D NMR spectroscopy were employed to determine their structures. The ability of the isolated compounds to inhibit NO production was also evaluated. Notably, compound **1** (IC_50_ = 14.10 ± 0.75 µM) effectively inhibited NO generation in LPS-stimulated RAW264.7 cells, demonstrating efficacy comparable to dexamethasone. To the best of our knowledge, this study represents the first investigation of chemical constituents isolated from the aerial parts of *G. formosum* and their pharmacological effects.

iNOS and COX-2 are key enzymes involved in the inflammatory response. They play crucial roles in the production of inflammatory mediators, such as NO and prostaglandins [34]. Therefore, targeting iNOS and COX-2 is a promising strategy for treating inflammatory diseases. Understanding the molecular mechanisms underlying the regulation of iNOS and COX-2 expression and activity is essential for developing effective therapeutic interventions to modulate inflammatory processes and mitigate related pathological conditions [35]. Drug development relies heavily on molecular docking technology, which analyzes the molecular behavior of target protein interactions. Molecular docking simulations were employed to examine the interactions and binding processes of compound **1** with the iNOS and COX-2 proteins. The results indicated that compound **1** could bind to the active sites of iNOS and COX-2 with binding energies of −11.5 and −8.7 kcal/mol, respectively, forming interactions with the key amino acid residues of these target proteins. Via evaluation in vitro and in silico, we hypothesize that gnetumoside A (**1**) selectively inhibits iNOS without affecting endothelial NO synthase (eNOS), thereby reducing inflammation while maintaining physiological NO production. This selective inhibition could provide an effective strategy for treating inflammatory diseases with minimal side effects.

Additionally, MD simulations were performed using a 50 ns trajectory to assess the conformational stability of the complexes formed by compound **1** with iNOS and COX-2. The simulations showed that compound **1** consistently occupied the active sites of both proteins, showing RMSD values of approximately 0.4 nm for iNOS and 0.3 nm for COX-2. Furthermore, RMSF values within the binding sites of both proteins exhibited minimal fluctuations, remaining below 0.2 nm, indicating that the complexes were stable throughout the 50 ns simulation period.

In conclusion, this study underscores the importance of natural product research in drug discovery and highlights the promising role of gnetumoside A (**1**) isolated from *G. formosum* in treating inflammation-related diseases. Future studies should focus on further validating its therapeutic benefits and exploring its potential in in vivo and clinical models.

## Figures and Tables

**Table 1:** ^1^H and ^13^C NMR data (MeOD-*d_4_*, 600 MHz) of compounds **1** and **2**.

Position	1		2	
	*δ*_C_	*δ*_H_ (*J* in Hz)	*δ*_C_	*δ*_H_ (*J* in Hz)
1	39.8, ^#^	1.13 (1H, m)/1.82 (1H, m)	39.7	1.13 (1H, m)/1.80 (1H, m)
2	30.1	1.60 (1H, m)/1.87 (1H, m)	35.2	2.17 (1H, m)/2.21 (1H, m)
3	79.3	3.53 (1H, m)	79.3	3.54 (1H, m)
4	39.8, ^#^	2.23 (1H, m)/2.36 (1H, m)	39.8	2.21 (1H, m)/2.37 (1H, m)
5	140.2	-	140.1	-
6	119.8	5.35 (1H, m)	119.8	5.37 (1H, t, *J* = 3.0, 4.8 Hz)
7	35.2	2.17 (1H, m)/2.22 (1H, m)	35.6	1.57 (1H, m)/2.25 (1H, m)
8	75.0	-	75.0	-
9	44.7	1.61 (1H, m)	44.7	-
10	38.1	-	38.0	-
11	26.1	1.67 (1H, m)/2.00 (1H, m)	26.1	1.68 (1H, m)/1.99 (1H, m)
12	75.3	4.86 (1H, m, H-12)	75.3	4.87 (1H, m)
13	57.8	-	57.7	-
14	89.6	-	89.6	-
15	34.5	1.97 (1H, m)/2.03 (1H, m)	34.5	1.89 (1H, m)/2.01 (1H, m)
16	34.1	1.96 (1H, m)/2.04 (1H, m)	34.0	1.90 (1H, m)/2.00 (1H, m)
17	88.6	-	88.5	-
18	11.3	1.64 (3H, s)	11.3	1.67 (3H, s)
19	18.5	1.12 (3H, s)	18.5 ^#^	1.14 (3H, s)
20	76.4	4.81 (1H, m)	76.4	4.82 (1H, m)
21	15.2	1.33 (3H, d, *J* = 6.0 Hz)	15.2	1.35 (3H, d, *J* = 6.6 Hz, H-21)
1′	131.7	-	131.7	-
2′/6′	131.0	7.96 (2H, dd, *J* = 1.2, 8.4 Hz)	131.0	7.98 (1H, dd, *J* = 1.2, 8.4 Hz)
3′/5′	129.8	7.35 (2H, m)	129.5	7.35 (1H, m)
4′	134.2	7.55 (1H, m)	134.2	7.57 (1H, m)
7′	167.0	-	167.0	-
1″	168.1	-	168.1	-
2″	120.1	6.07 (1H, d, *J* = 16.2 Hz)	120.1	6.09 (1H, d, *J* = 16.2 Hz)
3″	145.4	7.35 (1H, d, *J* = 16.2 Hz)	145.4	7.38 (1H, d, *J* = 16.2 Hz)
4″	135.6	-	135.6	-
5″/9″	129.2	7.24 (2H, t, *J* = 1.2, 8.4 Hz)	129.3	7.27 (2H, m)
6″/8″	129.5	7.34 (2H, m)	129.8	7.35 (2H)
7″	131.3	7.38(1H, m)	131.3	7.40 (1H, m)
CymI				
1‴	97.2	4.85 (1H, m)	97.2	4.89 (1H, m)
2‴	36.0	1.53 (1H, m)/2.16 (1H, m)	36.7	1.56 (1H, m)2.08 (1H, m)
3‴	79.2	3.60(1H, m)	78.6	3.86 (1H, m)
4‴	74.5	3.16 (1H, dd, *J* = 3.0, 9.6 Hz)	83.8	3.24 (1H, dd, *J* = 3.0, 9.6 Hz)
5‴	71.5	3.77 (1H, dd, *J* = 6.6, 9.6 Hz)	70.0	3.83 (1H, dd, *J* = 6.6, 9.6, Hz)
6‴	18.7	1.22 (3H, d, *J* = 6.6 Hz)	18.5 ^#^	1.21 (3H, t, *J* = 6.6 Hz)
3-OCH_3_	58.1	3.43 (3H, s, -OCH_3_)	58.1	3.47 (3H, #)
CymII				
1⁗			101.2	4.80 (1H, dd, *J* = 1.8, 9.6 Hz)
2⁗			35.6	1.57 (1H, m)/2.25 (1H, m)
3⁗			79.2	3.63 (1H, m)
4⁗			74.5	3.20 (1H, dd, *J* = 3.0, 9.6 Hz)
5⁗			71.3	3.74 (1H, dd, *J* = 6.0, 9.6 Hz)
6⁗			18.7	1.25 (3H, d, *J* = 6.6 Hz)
3-OCH_3_			58.5	3.48 (3H, m,)

^#^ Detected by HMBC spectrum; assignments were conducted by COSY, HSQC, HMBC, and NOESY experiments.

## Data Availability

Data are contained within the article and Supplementary Materials.

## Supplementary Materials

The following supporting information can be downloaded at https://www.mdpi.com/article/10.3390/plants13152100/s1. The details of NMR data and HR-ESI-MS data of new compounds **1** and **2** were reported. Figure S1: ^1^H-NMR spectrum (CD_3_OD, 600 MHz) of compound **1**; Figure S2: ^13^C-NMR spectrum (CD_3_OD, 150 MHz) of compound **1**; Figure S3: HSQC spectrum (CD_3_OD, 600 MHz) of compound **1**; Figure S4: COSY spectrum (CD_3_OD, 600 MHz) of compound **1**; Figure S5: HMBC spectrum (CD_3_OD, 600 MHz) of compound **1**; Figure S6: NOESY spectrum (CD_3_OD, 600 MHz) of compound **1**; Figure S7: HR-ESI-MS of compound **1**; Figure S8: ^1^H-NMR spectrum (CD_3_OD, 600 MHz) of compound **2**; Figure S9: ^13^C-NMR spectrum (CD_3_OD, 150 MHz) of compound **2**; Figure S10: COSY spectrum (CD_3_OD, 600 MHz) of compound **2**; Figure S11: HSQC spectrum (CD_3_OD, 600 MHz) of compound **2**; Figure S12: HMBC spectrum (CD_3_OD, 600 MHz) of compound **2**; Figure S13: NOESY spectrum (CD_3_OD, 600 MHz) of compound **2**; Figure S14: HR-ESI-MS of compound **2**.

## References

[1] Soares C.L.R., Wilairatana P., Silva L.R., Moreira P.S., Barbosa N.M.M.V., da Silva P.R., Coutinho H.D.M., de Menezes I.R.A., Felipe C.F.B. (2023). Biochemical aspects of the inflammatory process: A narrative review. Biomed. Pharmacother..

[2] Jiang H., Ji P., Shang X., Zhou Y. (2023). Connection between osteoarthritis and nitric oxide: From pathophysiology to therapeutic target. Molecules.

[3] Vinh L.B., Heo M., Phong N.V., Ali I., Koh Y.S., Kim Y.H., Yang S.Y. (2020). Bioactive compounds from *Polygala tenuifolia* and their inhibitory effects on lipopolysaccharide-stimulated pro-inflammatory cytokine production in bone marrow-derived dendritic cells. Plants.

[4] Duyen N.T., Vinh L.B., Phong N.V., Khoi N.M., Long P.Q., Hien T.T., Dat N.T., Lee K.Y. (2022). Steroid glycosides isolated from *Paris polyphylla* var. *chinensis* aerial parts and paris saponin II induces G1/S-phase MCF-7 cell cycle arrest. Carbohydr. Res..

[5] Liu Z., Vinh L.B., Tuan N.Q., Lee H., Kim E., Kim Y.-C., Sohn J.H., Yim J.H., Lee H.-J., Lee D.-S. (2023). Macrosphelides from antarctic fungus *Pseudogymnoascus* sp. (strain SF-7351) and their neuroprotective effects on BV2 and HT22 cells. Chem.-Biol. Interact..

[6] Tuan Anh H.L., Le Ba V., Do T.T., Phan V.K., Pham Thi H.Y., Bach L.G., Tran M.H., Tran Thi P.A., Kim Y.H. (2021). Bioactive compounds from *Physalis angulata* and their anti-inflammatory and cytotoxic activities. J. Asian Nat. Prod. Res..

[7] Ba Vinh L., Jang H.-J., Viet Phong N., Dan G., Won Cho K., Ho Kim Y., Young Yang S. (2019). Bioactive triterpene glycosides from the fruit of *Stauntonia hexaphylla* and insights into the molecular mechanism of its inflammatory effects. Bioorganic Med. Chem. Lett..

[8] Leonti M. (2013). Traditional medicines and globalization: Current and future perspectives in ethnopharmacology. Front. Pharmacol..

[9] Ernst E. (2005). The efficacy of herbal medicine—An overview. Fundam. Clin. Pharmacol..

[10] Prayoga D.K., Aulifa D.L., Budiman A., Levita J. (2024). Plants with anti-ulcer activity and mechanism: A review of preclinical and clinical studies. Drug Des. Dev. Ther..

[11] Stratton C.F., Newman D.J., Tan D.S. (2015). Cheminformatic comparison of approved drugs from natural product versus synthetic origins. Bioorganic Med. Chem. Lett..

[12] Iliya I., Tanaka T., Iinuma M., Ali Z., Furasawa M., Nakaya K.-i., Shirataki Y., Murata J., Darnaedi D. (2002). Stilbene derivatives from two species of Gnetaceae. Chem. Pharm. Bull..

[13] Do T.L. (2004). Vietnamese Medicinal Plants and Herbs.

[14] Vinh L.B., Han Y.K., Park S.Y., Kim Y.J., Phong N.V., Kim E., Ahn B.-G., Jung Y.W., Byun Y., Jeon Y.H. (2023). Identification of triterpenoid saponin inhibitors of interleukin (IL)-33 signaling from the roots of *Astragalus membranaceus*. J. Funct. Foods.

[15] Cao T.Q., Phong N.V., Kim J.H., Gao D., Anh H.L.T., Ngo V.-D., Vinh L.B., Koh Y.S., Yang S.Y. (2021). Inhibitory effects of cucurbitane-type triterpenoids from *Momordica charantia* fruit on lipopolysaccharide-stimulated pro-inflammatory cytokine production in bone marrow-derived dendritic cells. Molecules.

[16] Nguyen T.M.N., Le H.S., Le B.V., Kim Y.H., Hwang I. (2020). Anti-allergic effect of inotodiol, a lanostane triterpenoid from chaga mushroom, via selective inhibition of mast cell function. Int. Immunopharmacol..

[17] Li X., Sun H., Ye Y., Chen F., Tu J., Pan Y. (2005). Three new immunomodulating C21-steroidal glycosides from the stems of *Stephanotis mucronata*. Chem. Biodivers..

[18] Chen H., Xu N., Zhou Y., Qiao L., Cao J., Yao Y., Hua H., Pei Y. (2008). Steroidal glycosides from the roots of *Cynanchum amplexicaule* Sieb. et Zucc. Steroids.

[19] Wang Y.-B., Su S.-S., Tang M.-X., Zhao D., Chen G., Chen S.-F., Wang H.-F., Pei Y.-H. (2021). Two new pregnane steroidal glycosides from *Cynanchum taihangense*. Nat. Prod. Res..

[20] Kubo S., Kuroda M., Yokosuka A., Sakagami H., Mimaki Y. (2015). Amurensiosides L-P, five new cardenolide glycosides from the roots of *Adonis amurensis*. Nat. Prod. Commun..

[21] Cioffi G., Sanogo R., Vassallo A., Dal Piaz F., Autore G., Marzocco S., De Tommasi N. (2006). Pregnane glycosides from *Leptadenia pyrotechnica*. J. Nat. Prod..

[22] Kanwar J.R., Kanwar R.K., Burrow H., Baratchi S. (2009). Recent advances on the roles of no in cancer and chronic inflammatory disorders. Curr. Med. Chem..

[23] Phong N.V., Anh D.T.N., Chae H.Y., Yang S.Y., Kwon M.J., Min B.S., Kim J.A. (2022). Anti-inflammatory activity and cytotoxicity against ovarian cancer cell lines by amide alkaloids and piperic esters isolated from *piper longum* fruits: In vitro assessments and molecular docking simulation. Bioorganic Chem..

[24] Vinh L.B., Nguyet N.T.M., Ye L., Dan G., Phong N.V., Anh H.L.T., Kim Y.H., Kang J.S., Yang S.Y., Hwang I. (2020). Enhancement of an in vivo anti-inflammatory activity of oleanolic acid through glycosylation occurring naturally in *Stauntonia hexaphylla*. Molecules.

[25] Fischmann T.O., Hruza A., Niu X.D., Fossetta J.D., Lunn C.A., Dolphin E., Prongay A.J., Reichert P., Lundell D.J., Narula S.K. (1999). Structural characterization of nitric oxide synthase isoforms reveals striking active-site conservation. Nat. Struct. Mol. Biol..

[26] Orlando B.J., Malkowski M.G. (2016). Crystal structure of rofecoxib bound to human cyclooxygenase-2. Acta Crystallogr. Sect. F.

[27] Laskowski R.A., Swindells M.B. (2011). Ligplot+: Multiple ligand–protein interaction diagrams for drug discovery. J. Chem. Inf. Model..

[28] Abraham M.J., Murtola T., Schulz R., Páll S., Smith J.C., Hess B., Lindahl E. (2015). GROMACS: High performance molecular simulations through multi-level parallelism from laptops to supercomputers. SoftwareX.

[29] Van Der Spoel D., Lindahl E., Hess B., Groenhof G., Mark A.E., Berendsen H.J.C. (2005). GROMACS: Fast, flexible, and free. J. Comput. Chem..

[30] Lee J., Cheng X., Swails J.M., Yeom M.S., Eastman P.K., Lemkul J.A., Wei S., Buckner J., Jeong J.C., Qi Y. (2016). CHARMM-GUI input generator for NAMD, GROMACS, AMBER, OpenMM, and CHARMM/OpenMM simulations using the CHARMM36 additive force field. J. Chem. Theory Comput..

[31] Tariq L., Bhat B.A., Hamdani S.S., Mir R.A., Aftab T., Hakeem K.R. (2021). Phytochemistry, pharmacology and toxicity of medicinal plants. Medicinal and Aromatic Plants: Healthcare and Industrial Applications.

[32] Thomford N.E., Senthebane D.A., Rowe A., Munro D., Seele P., Maroyi A., Dzobo K. (2018). Natural products for drug discovery in the 21st century: Innovations for novel drug discovery. Int. J. Mol. Sci..

[33] Atanasov A.G., Zotchev S.B., Dirsch V.M., Supuran C.T. (2021). Natural products in drug discovery: Advances and opportunities. Nat. Rev. Drug Discov..

[34] Pradhan P., Vijayan V., Liu B., Martinez-Delgado B., Matamala N., Nikolin C., Greite R., DeLuca D.S., Janciauskiene S., Motterlini R. (2024). Distinct metabolic responses to heme in inflammatory human and mouse macrophages–role of nitric oxide. Redox Biol..

[35] Cock I.E. (2024). *Terminalia ferdinandiana* Exell. extracts reduce pro-inflammatory cytokine and PGE_2_ secretion, decrease COX-2 expression and down-regulate cytosolic NF-κB levels. Inflammopharmacology.

