# Supplementary material for: Two New Steroidal Saponins with Potential Anti-Inflammatory Effects from the Aerial Parts of Gnetum formosum Markgr
Source: Plants (Basel). 2024 Jul 29;13(15):2100. doi: 10.3390/plants13152100 (PMC11314289; doi:10.3390/plants13152100)
Supplement: Supplementary file 1 [file plants-13-02100-s001.zip › plants-3106295-supplementary.pdf]

# SUPPLEMENTARY MATERIAL

# Two New Steroidal Saponins with Potential Anti-Inflammatory Effects from the Aerial Parts of *Gnetum formosum* Markgr.

**Ngo Van Hieu [1,2,†], Le Ba Vinh [3,†], Nguyen Viet Phong [3,4], Pham Van Cong [1], Nguyen Tien Dat [1], Nguyen Van Dan [1], Ngo Viet Duc [1], Hoang Minh Tao [1], Le Thi Tam [1], Le Tuan Anh [5], Nguyen Cao Cuong [6], Bui Huu Tai [3], Seo Young Yang [4,*] and Hoang Le Tuan Anh [1,2,*]**

[1] Center for High Technology Research and Development, Vietnam Academy of Science and Technology (VAST), Hanoi 10072, Vietnam; ngohieu03041997@gmail.com (N.V.H.); phamcong1990@gmail.com (P.V.C.); ngtiend@gmail.com (N.T.D.); nvdan100@gmail.com (N.V.D.); ngovietduc.cretech@gmail.com (N.V.D.); htm1205@gmail.com (H.M.T.); lamtamhoaduoc@gmail.com (L.T.T.)

[2] Graduate University of Science and Technology, Vietnam Academy of Science and Technology (VAST), Hanoi 10072, Vietnam

[3] Institute of Marine Biochemistry, Vietnam Academy of Science and Technology (VAST), Hanoi 10072, Vietnam; vinhrooney@gmail.com (L.B.V.); ngvietphong@gmail.com (N.V.P.); bhtaiich@gmail.com (B.H.T.)

[4] Department of Biology Education, Teachers College and Institute for Phylogenomics and Evolution, Kyungpook National University, Daegu 41566, Republic of Korea

[5] Vietnam National Museum of Nature, Vietnam Academy of Science and Technology (VAST), Hanoi 10072, Vietnam; tasa207@gmail.com

[6] Faculty of Medicine and Pharmacy, Yersin University, Da Lat 66100, Vietnam nguyencaocuong2712@gmail.com

* Correspondence: syy@knu.ac.kr (S.Y.Y.); hltanh@ctctt.vast.vn (H.L.T.A.); Tel.: +82-53-950-5910 (S.Y.Y.); +84-849-4815-1838 (H.L.T.A.); Fax: +82-53-950-6809 (S.Y.Y.)

† These authors contributed equally to this paper.

**Abstract:** *Gnetum formosum* Markgr., a member of the Gnetaceae family, is distributed in Vietnam. This plant remains a botanical enigma with an unexplored diversity of chemical constituents and pharmacological effects. In this study, two new steroidal saponins, namely gnetumosides A (**1**) and B (**2**), were isolated from the aerial parts of *G. formosum*. Their chemical structures were elucidated using spectroscopic techniques, including high-resolution electrospray ionization mass spectrometry (HR-ESI-MS) and NMR, along with chemical hydrolysis and comparison with the reported literature. The potential anti-inflammatory effects of the isolated compounds were evaluated by measuring lipopolysaccharide-stimulated nitric oxide (NO) production in murine macrophage cells. Notably, compound **1** exhibited the most potent inhibitory activity ($IC_{50}$ = 14.10 ± 0.75 μM), comparable to dexamethasone. Additionally, the mechanisms underlying the observed anti-inflammatory effects were investigated through molecular docking and molecular dynamics simulations on inducible nitric oxide synthase (iNOS) and cyclooxygenase-2 (COX-2) proteins. This study is the first to investigate the chemical constituents and pharmacological effects of *G. formosum*.

**Keywords:** *Gnetum formosum* Markgr.; steroidal saponin; gnetumoside A; gnetumoside B; anti-inflammatory

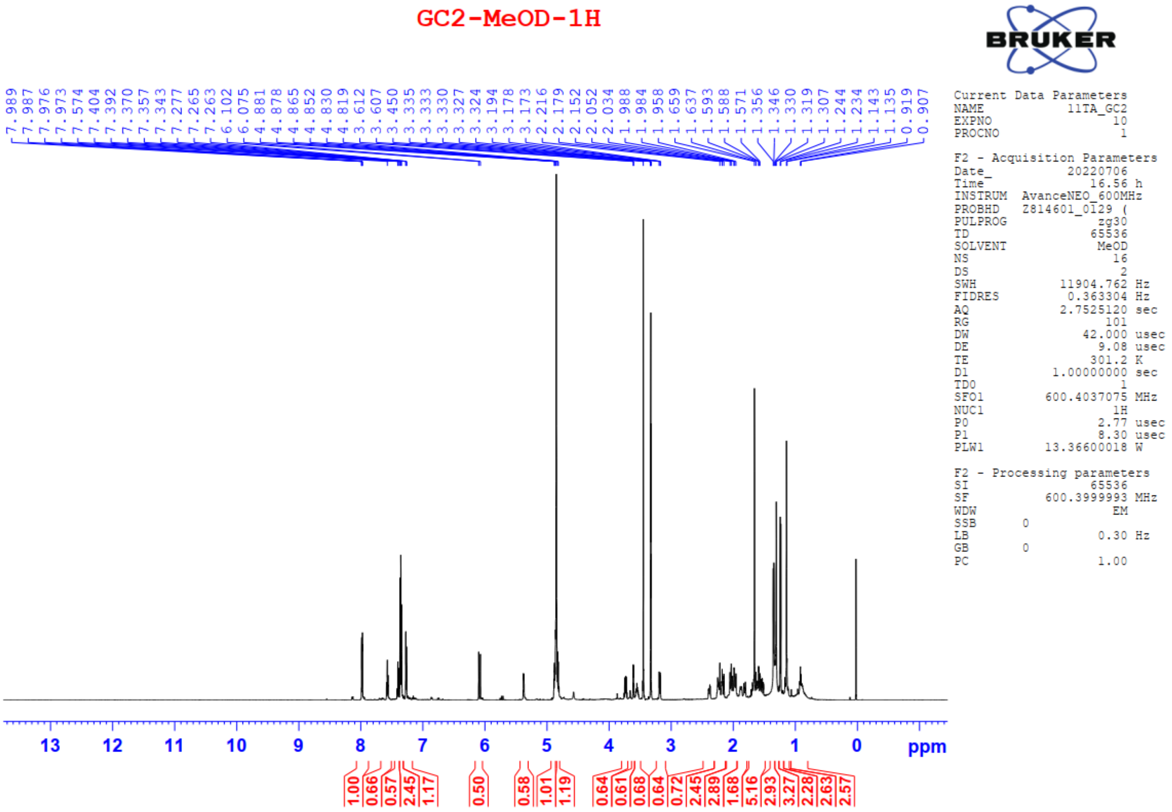


**Figure S1.** $^{1}H$-NMR spectrum ($CD_3OD$, 600 MHz) of compound **1**

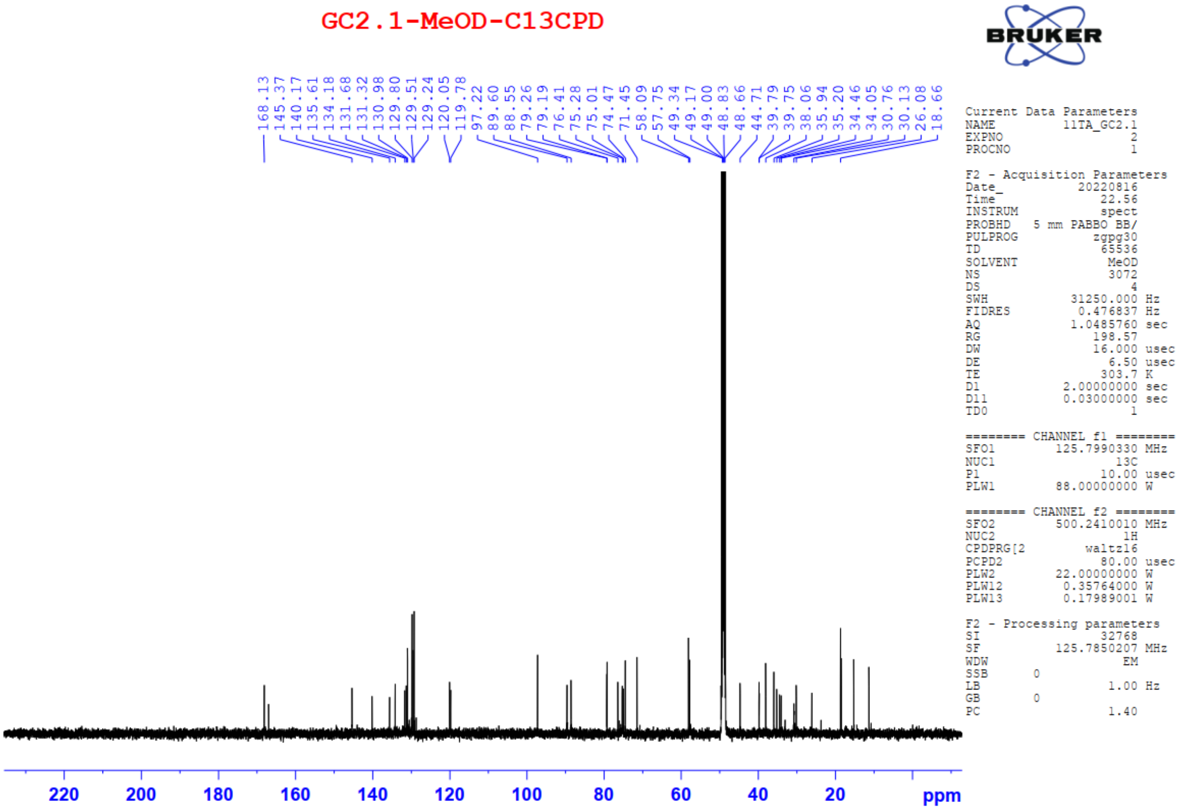


**Figure S2.** $^{13}C$-NMR spectrum ($CD_3OD$, 150 MHz) of compound **1**

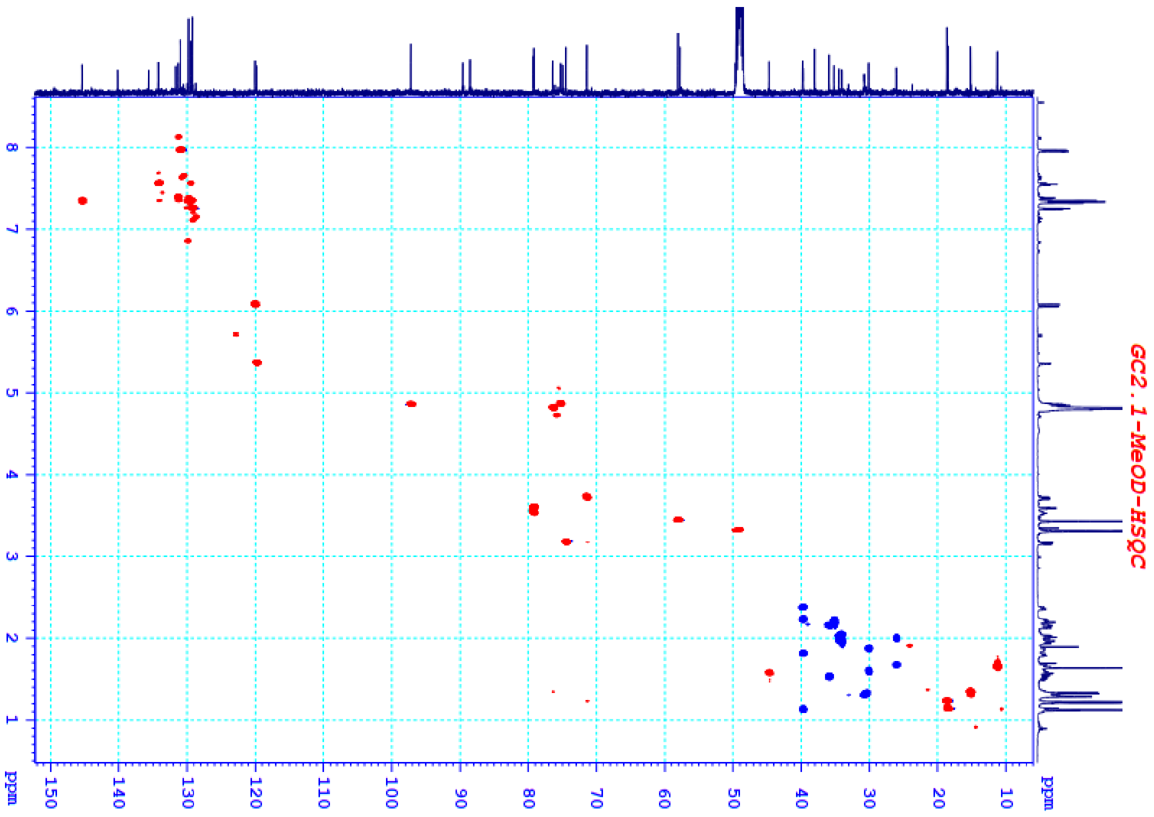


**Figure S3.** HSQC spectrum ($CD_3OD$, 600 MHz) of compound **1**

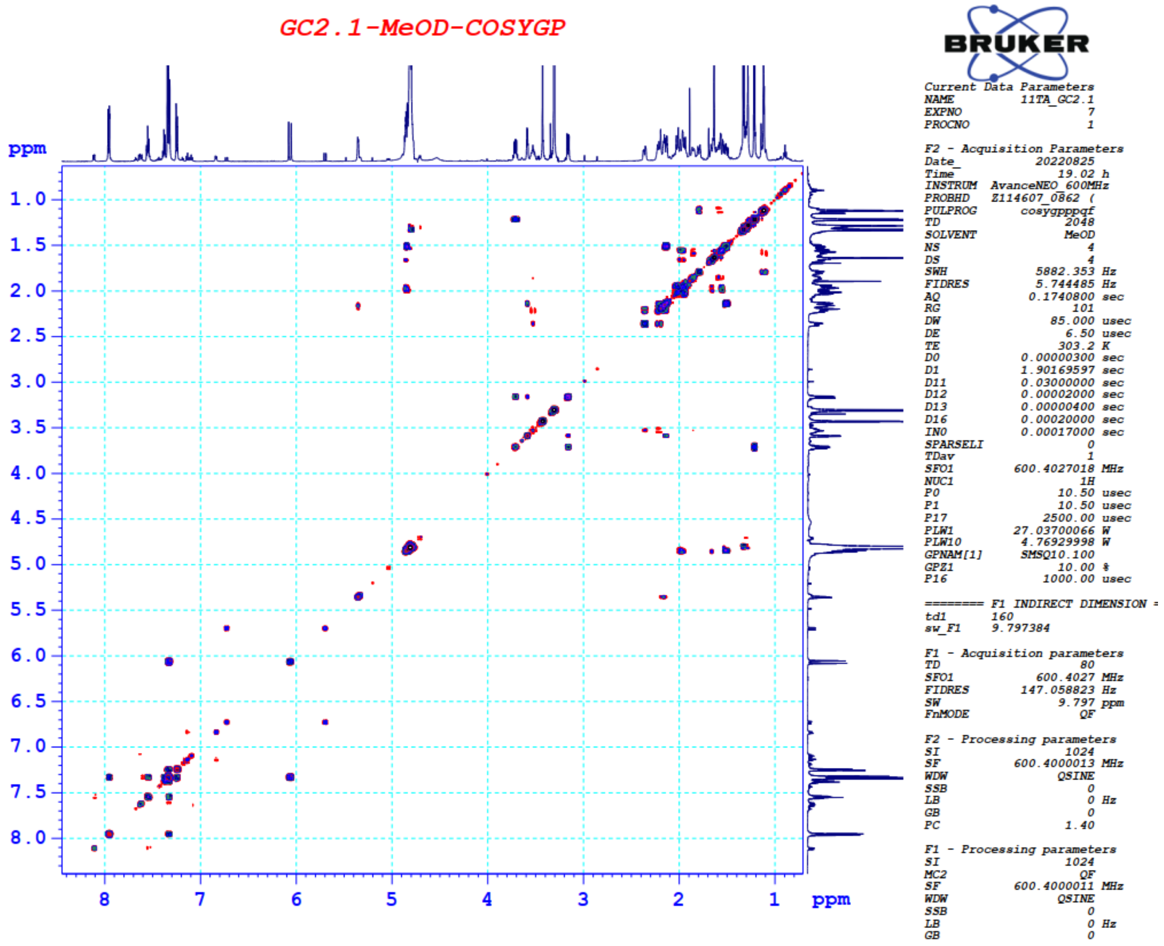


**Figure S4.** COSY spectrum ($CD_3OD$, 600 MHz) of compound **1**

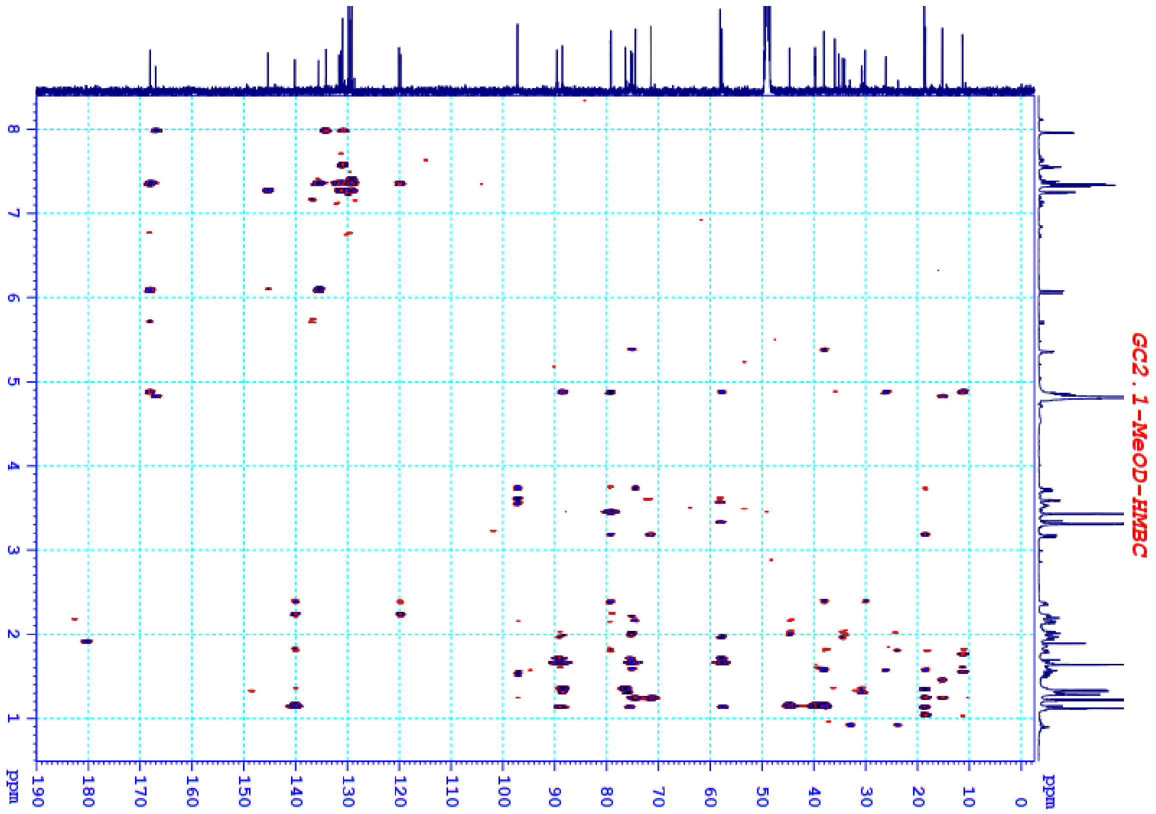


**Figure S5.** HMBC spectrum ($CD_3OD$, 600 MHz) of compound **1**

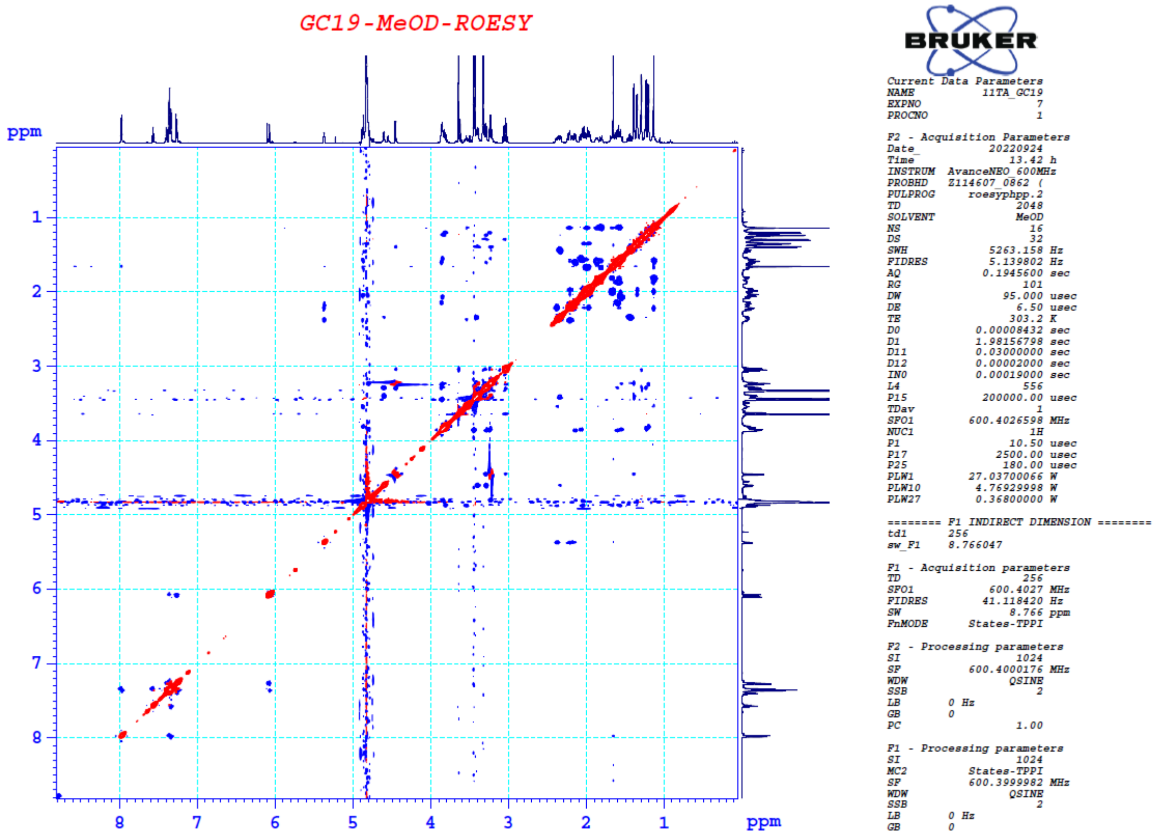


**Figure S6.** NOESY spectrum ($CD_3OD$, 600 MHz) of compound **1**

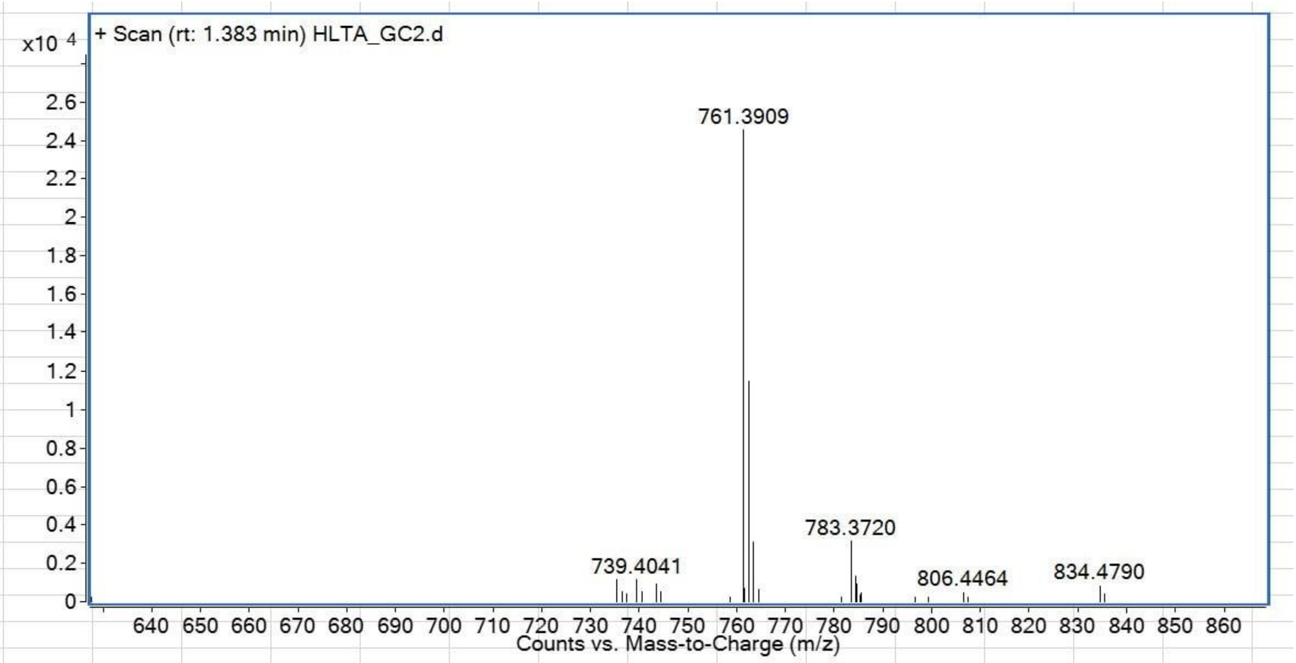


**Figure S7.** HR-ESI-MS of compound **1**

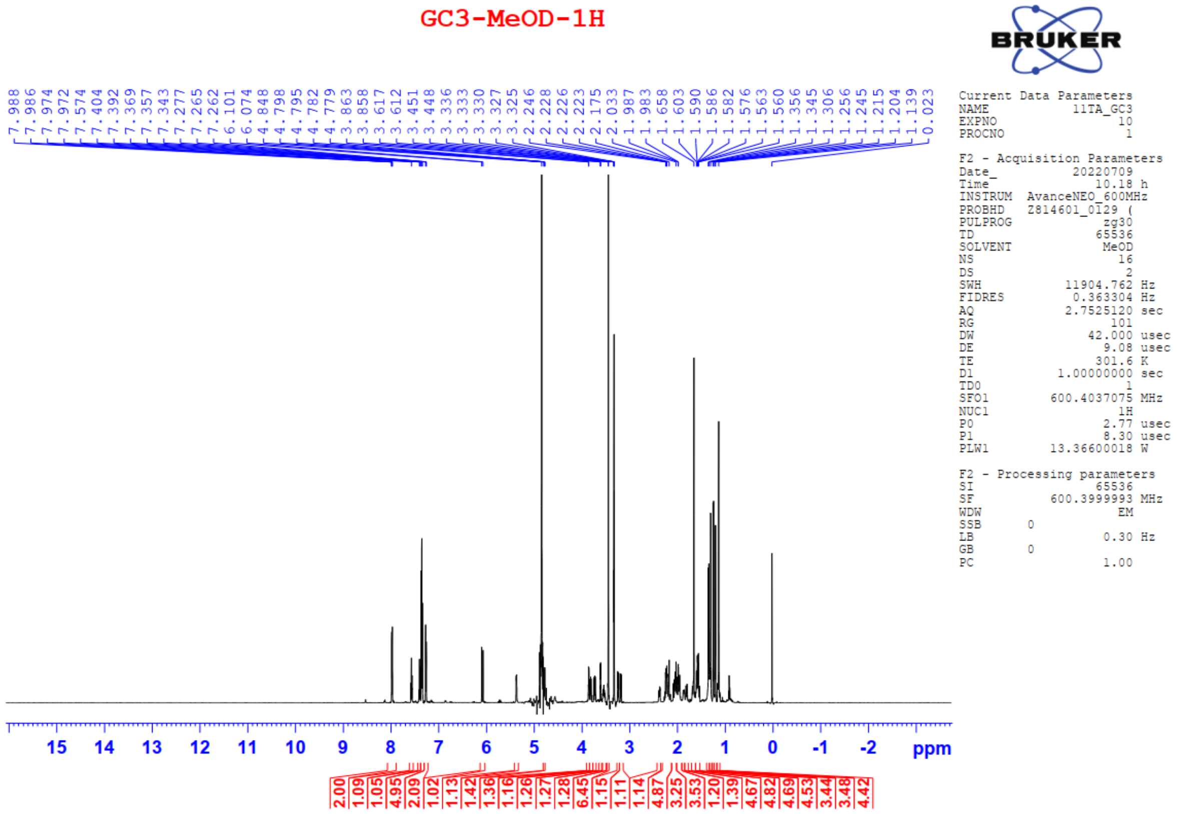

**Figure S8.** $^{1}$H-NMR spectrum ($CD_3OD$, 600 MHz) of compound **2**

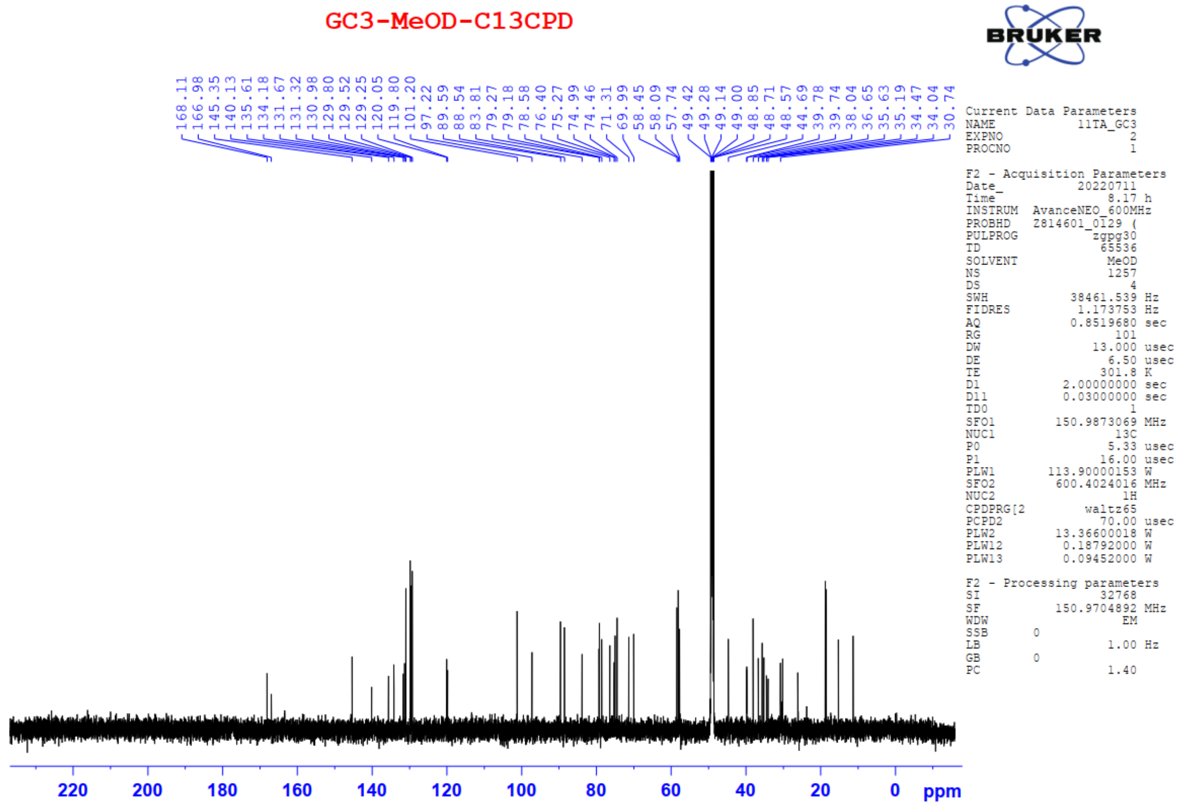

**Figure S9.** $^{13}$C-NMR spectrum ($CD_3OD$, 150 MHz) of compound **2**

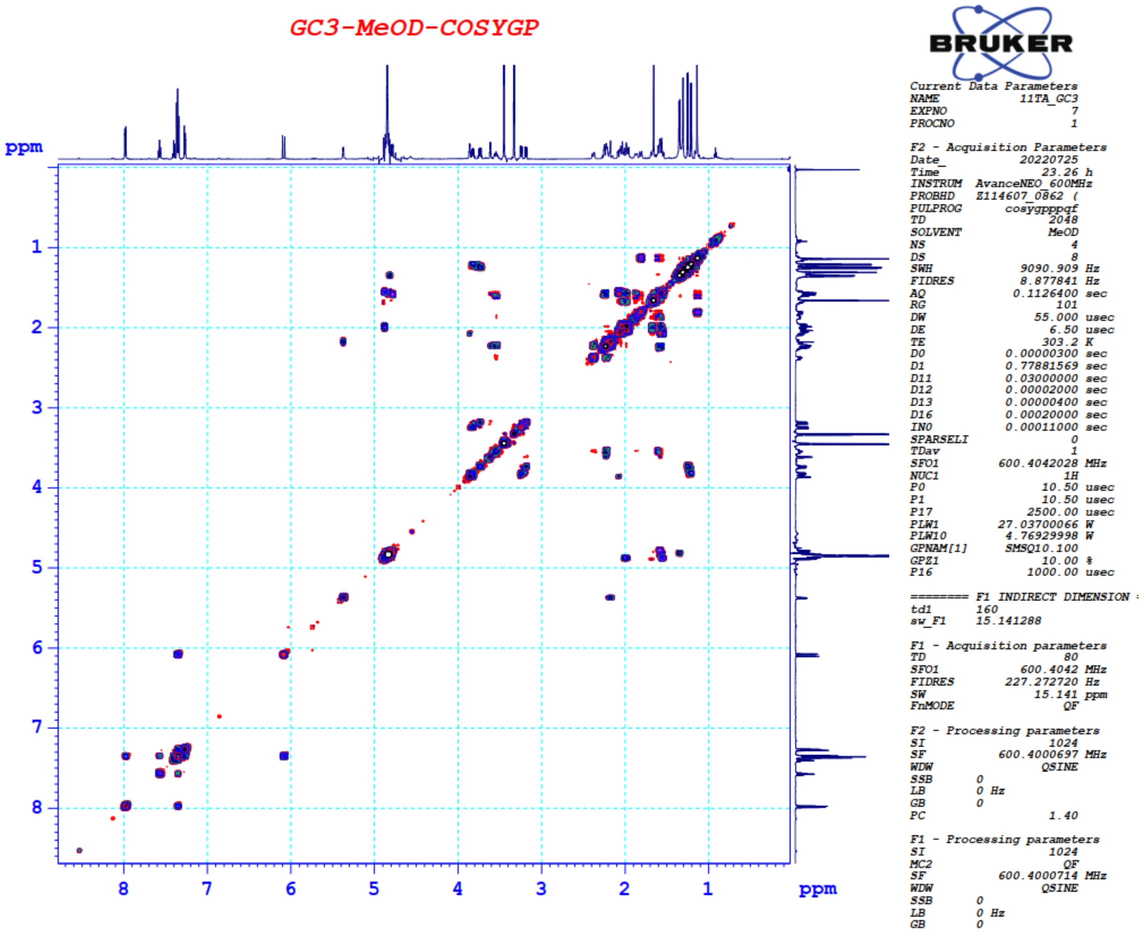


**Figure S10.** COSY spectrum ($CD_3OD$, 600 MHz) of compound **2**

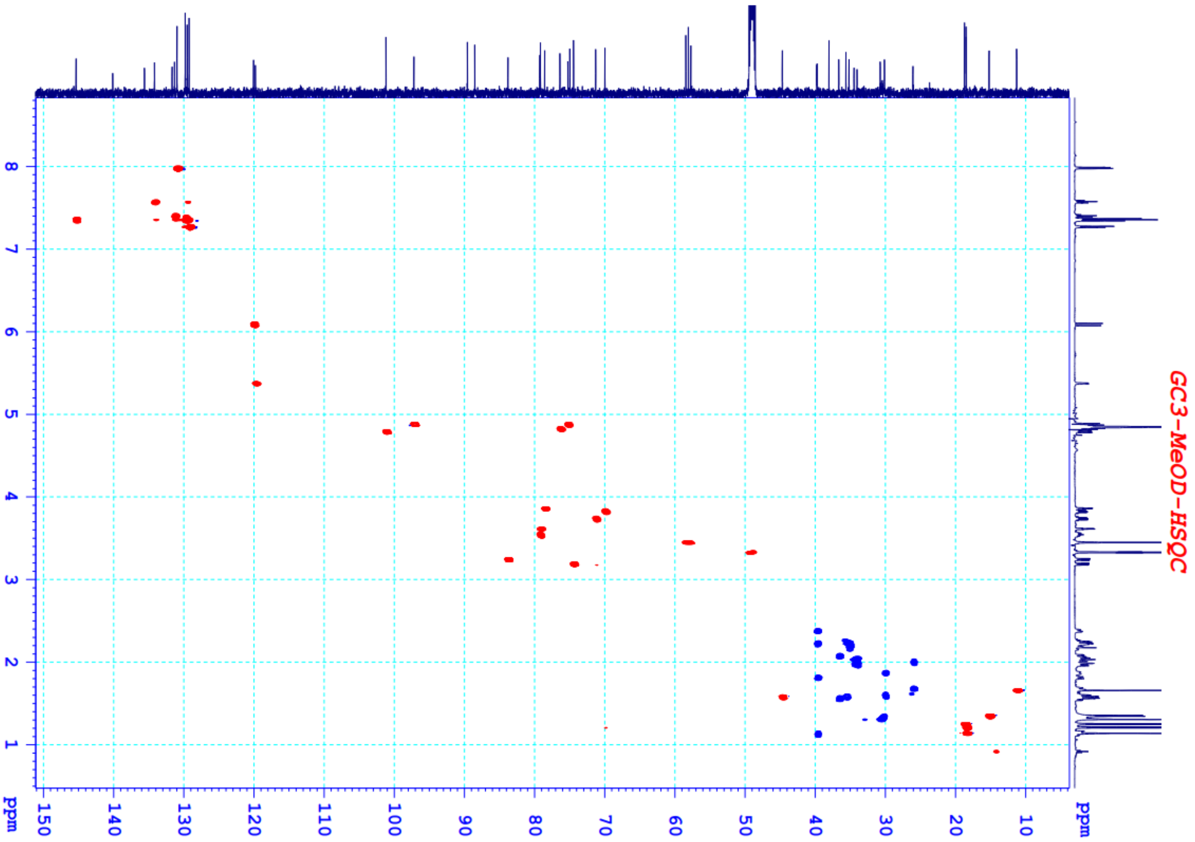


**Figure S11.** HSQC spectrum ($CD_3OD$, 600 MHz) of compound **2**

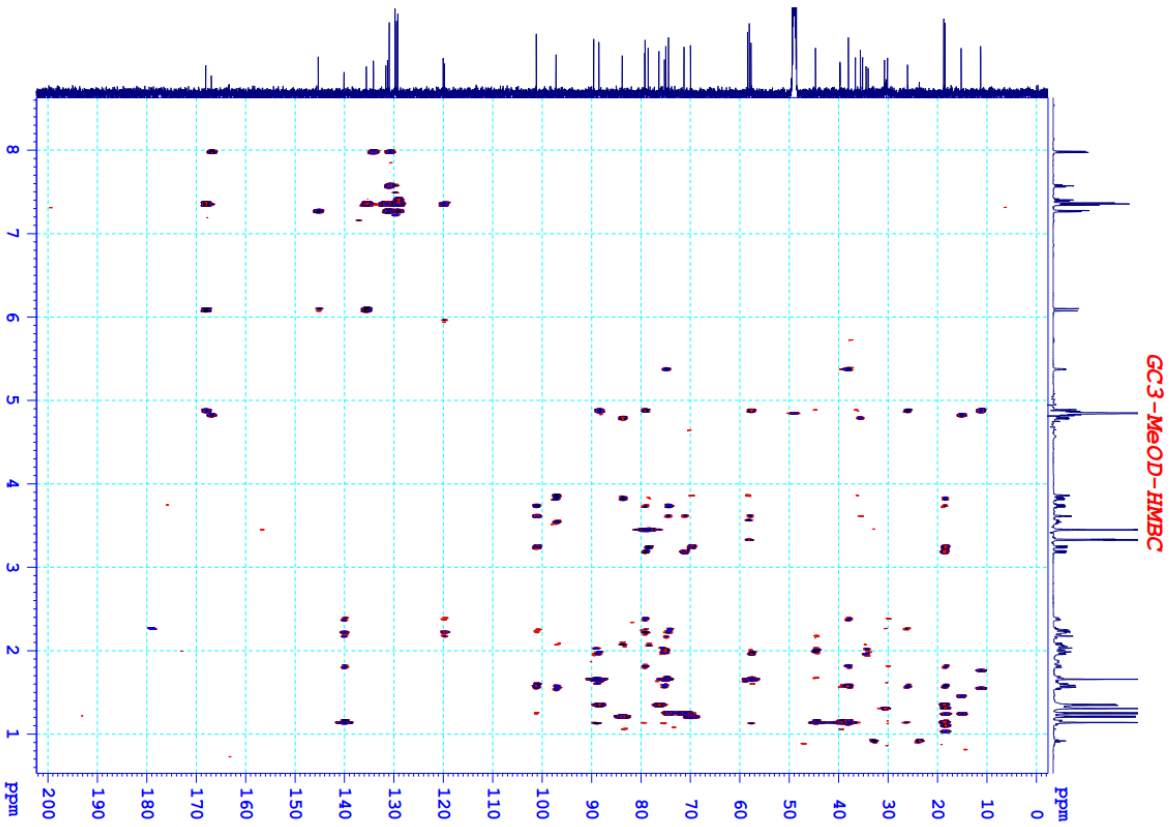


**Figure S12.** HMBC spectrum ($CD_3OD$, 600 MHz) of compound **2**

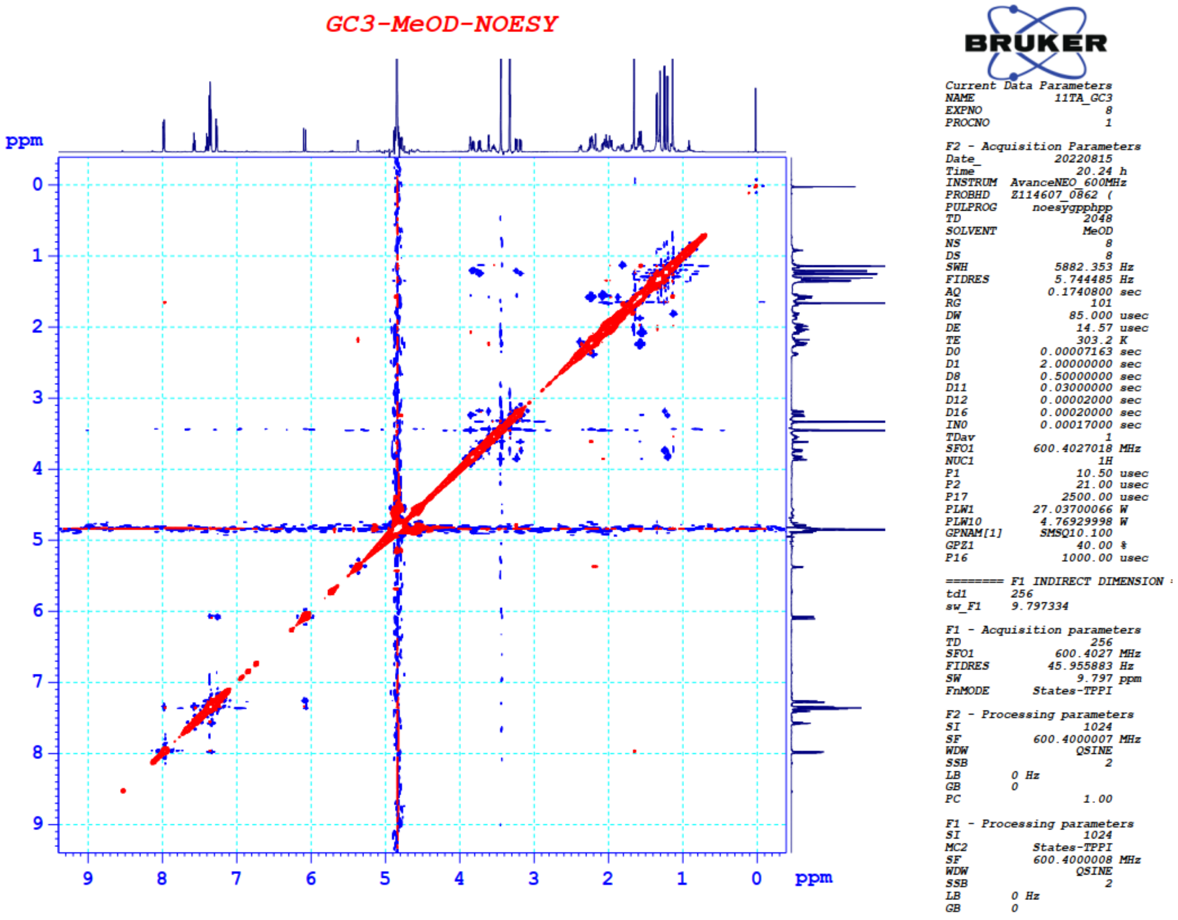


**Figure S13.** NOESY spectrum ($CD_3OD$, 600 MHz) of compound **2**

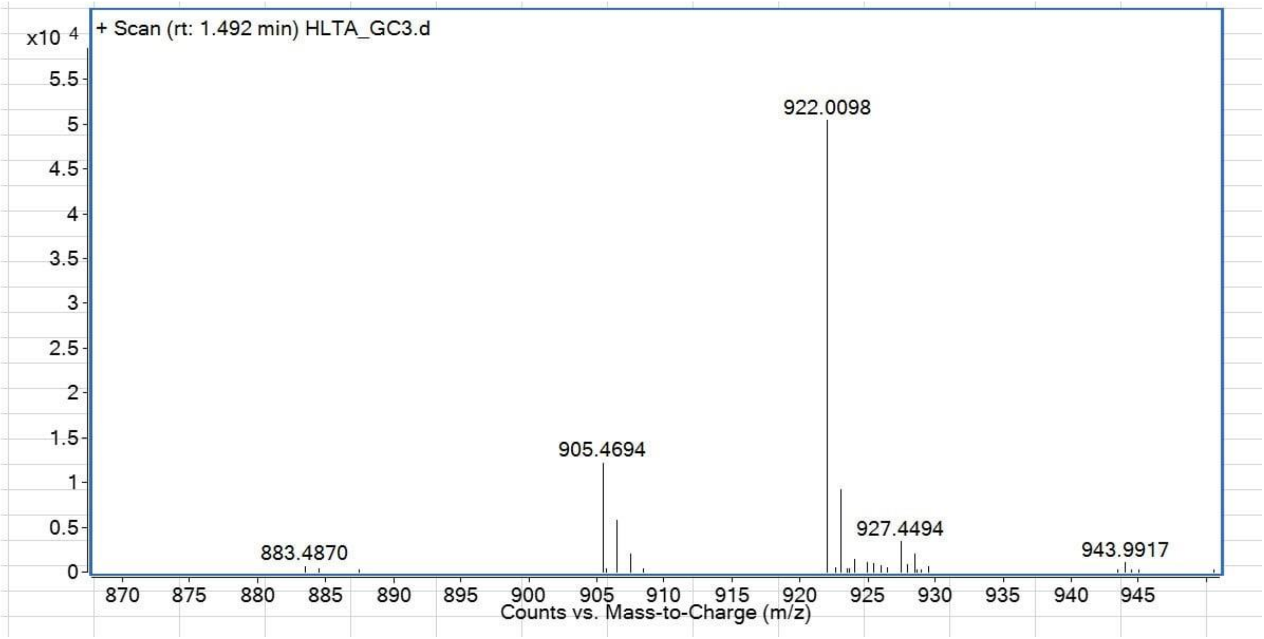


**Figure S14.** HR-ESI-MS of compound **2**
